# Possible manufacture of test allergens in public pharmacies for the diagnosis of type I allergies: Legal aspects 

**DOI:** 10.5414/ALX02514E

**Published:** 2024-07-22

**Authors:** Robin Jost, Sabine Kespohl, Kathrin E. Paulus-Tremel, Julia Zimmer, Andreas Bonertz, Ingrid Sander, Thomas Klose, Lena-Maria Altin, Simone Heller, Ralph Heimke-Brinck, Frank Dörje, Susanne Philippus, Matthias Meyer, Sabrina Segebrecht, Torsten Wessel, Dieter Starke, Stefan Schülke, Monika Raulf, Vera Mahler

**Affiliations:** 1 Allergology Division, Paul-Ehrlich-Institut (PEI), Langen (Hesse),; 2 Friedrich-Alexander-Universität Erlangen-Nürnberg (FAU), Erlangen,; 3 Institute for Prevention and Occupational Medicine of the German Social Accident Insurance (DGUV), Institute of the Ruhr-Universität Bochum (IPA), Bochum,; 4 Sonnenschein Apotheke, Koblenz,; 5 Pharmacy Department, Erlangen University Hospital, Erlangen,; 6 Thuringian State Authority for Consumer Protection, Department Pharmacy, Bad Langensalza,; 7 Bundeswehr Commissioner for the Surveillance of Medicinal Products, Supervisory Agency for Public Law Tasks of the Bundeswehr Medical Service South, Munich,; 8 Drug Supervision, State Social Services Agency of Land Schleswig-Holstein, Neumünster,; 9 Pharmacy Inspector, Local inspectorate for pharmacies, District of Wesel, Moers, and; 10 State Agency for Social Affairs, Youth and Care, Rhineland-Palatinate, Koblenz, Germany,; * Current address: University of Bayreuth (UBT), Kulmbach, Germany

**Keywords:** skin prick testing, occupational allergy, finished medicinal product, marketing authorization, diagnostic gap, pharmacy manufacturing, German Medicinal Products Act (AMG), European Pharmacopoeia (Ph. Eur.), Ordinance on the Operation of Pharmacies (ApBetrO), quality assurance

## Abstract

The availability of high-quality skin test allergens is a prerequisite for the reliable diagnosis of occupational type I allergies. Due to the withdrawal of existing marketing authorizations (MAs) by pharmaceutical companies and the lack of new MAs for commercial test allergens, there is an increasing diagnostic gap in Germany and other EU member states, which makes it necessary to investigate alternative ways of providing in vivo diagnostics. The German Medicinal Products Act (Arzneimittelgesetz = AMG) allows for the possibility of preparing medicinal products in pharmacies without the need for an MA or a manufacturing authorization pursuant to Section 13 (2) No. 1 in conjunction with Section 13 (2a) Sentence 2 No. 3 AMG. This also includes test allergens. In addition to the AMG, the requirements of the German Ordinance on the Operation of Pharmacies (Apothekenbetriebsordnung – ApBetrO) and the European Pharmacopoeia apply in particular. Medicolegal and practical challenges, as well as potentials of manufacturing skin prick test solutions in public pharmacies are presented based on examples of different allergen source materials.

## Introduction 

Skin prick testing in combination with clinical history is an important part of confirming the diagnosis of IgE-mediated type I allergies. According to the German Medicinal Products Act (Arzneimittelgesetz = AMG) [1] and European Directive 2001/83/EC [2] test allergens are medicinal products requiring marketing authorization. 

In recent years, the availability of authorized commercial test allergens for the detection of allergies to less common allergen sources (e.g., occupational allergens) has decreased considerably. This is the result of constrained product portfolios of pharmaceutical companies due to active withdrawal of marketing authorizations by the marketing authorization holders or expiry of marketing authorizations through the so-called “sunset clause” ^1^. This affects skin prick test solutions, intradermal test solutions, nasal and conjunctival provocation solutions as well as patch test substances [3]. Reasons given for this reduction are a low demand combined with high costs associated with the manufacturing and regulatory processes. In addition, there is scarce activity on the part of allergen manufacturers with regard to new marketing authorization applications for rare test allergens. Similar constraints have been reported in other EU member states [4]. Activities to harmonize regulatory procedures concerning allergen products at an European level have been addressed elsewhere [4, 5, 6]. 

^1^Sunset clause: According to section 31 sub-section 1 No. 1 AMG [1], the marketing authorization expires if the authorized medicinal product is not placed on the market within 3 years of the authorization being granted or if the authorized medicinal product that was placed on the market after the authorization was granted is no longer on the market for 3 consecutive years. 

Quality and product-specific standardization of test allergens are essential for diagnostic validity [7]. In the past, it has been shown that available test allergens in some cases feature quality deficiencies, which are associated with a low sensitivity of the skin prick tests performed [8]. 

In order to support the maintenance of authorized test allergens on the German market, the Paul-Ehrlich-Institut (PEI), in coordination with the Federal Ministry of Health (BMG), grants a fee reduction down to 25% for all regulatory procedures (official batch testing, variations, scientific advice, marketing authorization) in connection with rare test allergens upon informal request by the pharmaceutical manufacturer. Nevertheless, there is currently a diagnostic gap for numerous occupational allergens (see article by Raulf & Kespohl in part 1 of the special issue [9]). If activities by various stakeholders (scientific medical societies, doctors’ and patients’ associations, statutory accident insurance institutions, etc.) are not successful in keeping commercial in vivo diagnostics for less common test allergens available on the market, pharmacy manufacturing could be an alternative. This article examines the possible manufacture of test allergens in public pharmacies for the diagnosis of type I allergies, taking into account the legal requirements. 

## Legal framework for the manufacturing of skin prick test solutions in pharmacies 

The German AMG generally permits the manufacture of test allergens by a pharmacy without a manufacturing authorization pursuant to Section 13 (2) No. 1 in conjunction with Section 13 (2a) sentence 2 No. 3 AMG within the scope of normal pharmacy operations [1]. 

The general legal requirements for medicinal products and the special requirements for allergen products according to the monographs of the European Pharmacopoeia (Ph. Eur.) as well as the legal pharmacy requirements for manufacture must be taken into account and harmonized with each other. 

Although permitted under the AMG [1], the preparation of test allergens in pharmacies is not (yet) established at present. The specifications defined in the German Ordinance on the Operation of Pharmacies (Apothekenbetriebsordnung = ApBetrO) [10] must be observed for manufacture in pharmacies. Of particular importance here are Section 6 (“General Regulations Governing Preparation and Testing”) and Section 11 (“Primary Substances”) of the ApBetrO, which deal with testing obligations in pharmacies. According to Section 1a (6) ApBetrO, a primary substance is each compound or preparation of compounds used in the production of a pharmaceutical with the exception of packaging materials. This is to be distinguished from the term active pharmaceutical ingredient (API) starting material, which is defined as “a raw material, intermediate, or an active substance that is used in the production of an active substance and that is incorporated as a significant structural fragment into the structure of the active substance” [11] (e.g., in the case of the production of a skin prick test for the diagnosis of cod allergy, the powdered, defatted cod would be the starting material, the API would be the protein-containing crude extract obtained from it) [11]. Principally, medicinal products and their primary substances must be tested with regard to identity and quality (content, purity) according to recognized pharmaceutical rules (e.g., pharmacopoeia monographs, German Drug Codex/New German Formulary – (Deutscher Arzneimittel-Codex/Neues Rezeptur Formularium = DAC/NRF)). In the Ph. Eur. monograph “Allergen products” (1063) [12] the following methods for identity testing are mentioned:

- The protein profile of an in-house reference preparation is compared with that of the sample to be analyzed.
- Suitable methods for creating the protein profile include isoelectric focusing, SDS-PAGE, immunoelectrophoresis, immunoblotting, liquid chromatography or mass spectrometry.
- In exceptional cases where no internal reference preparation is available, a representative batch of the preparation may be used.

According to Section 11 (1) ApBetrO [10], only primary substances whose adequate quality has been determined may be used for the preparation of pharmaceuticals. The regulations of Section 6 (1) and (3) shall apply accordingly to the testing of primary substances. If primary substances are purchased whose quality is proven by a test certificate pursuant to Section 6 (3), at least their identity must be ascertained at the pharmacy (Section 11 (2) ApBetrO [10]). 

Section 6 (3) ApBetrO [10] specifies which facilities are authorized to issue test certificates:

- Facilities that have been granted a national or a European manufacturing authorization
- A facility that has been granted a license pursuant to Section 1 (2) in connection with Section 2 of the German Pharmacies Act (Apothekengesetz = ApoG) [13] (i.e., public main and branch pharmacies)
- Experts within the meaning of Section 65 (4) AMG [1]

For certification, a manufacturing authorization according to Section 13 AMG [1] or – for facilities in a member state of the European Union or the European Economic Area – in accordance with Article 40 of Directive 2001/83/EC [2] is required. However, public pharmacies are exempt from the need for a manufacturing authorization and are listed in Section 6 (3) No. 3 ApBetrO [10] explicitly (a facility that has been granted a license pursuant to Section 1 (2) in connection with Section 2 of the German Pharmacies Act [13]). The same applies to experts within the meaning of Section 65 (4) AMG, where no manufacturing authorization is required either. In the case of active substances, proof of good manufacturing practice (GMP)-compliant manufacture would also be required in accordance with Section 11 (2) sentence 2 ApBetrO; however, this is not required for allergen starting materials that are still upstream of the active substance stage. 

### Extemporaneous preparations (“Rezeptur” according to ApBetrO [10])/Preparations of Pharmaceuticals to be kept in stock in larger quantities (“Defektur” according to ApBetrO [10]) 

The ApBetrO distinguishes between two different types of manufacture: the extemporaneous preparation (“Rezeptur”) according to Section 7 and preparation of pharmaceuticals to be kept in stock in larger quantities (“Defektur”) according to § 8 ApBetrO [10]. The difference is the time of manufacture: an extemporaneous preparation is manufactured subsequently to an individual prescription, while a preparation of pharmaceuticals to be kept in stock may be manufactured in advance for a maximum of 100 packs per day, provided that in compliance with Section 21 (2) AMG [1] there is evidence of frequent medical prescriptions. Accordingly, the requirements for the release of the manufactured medicinal products also differ: In the case of extemporaneous preparations, analytical testing of the manufactured medicinal product can be omitted, provided that the quality can be guaranteed by the manufacturing process and the in-process controls as well as the organoleptic testing (appearance, texture, color, odor). Preparations of pharmaceuticals to be kept in stock, on the other hand, must always undergo final testing (Section 8 (3) and (4) ApBetrO). Appropriate test instructions must be prepared for this purpose, whereby the final choice of test criteria is the responsibility of the pharmacist. The test must be suitable for demonstrating the quality of the medicinal product. In the case of allergen products, e.g., testing the protein content (using the Bradford assay) and/or the total protein profile (using sodium dodecyl sulphate polyacrylamide gel electrophoresis (SDS-PAGE)) would be suitable. Depending on the allergen source material, testing for sterility, microbial contamination, or allergenic activity may also be necessary. Pharmacies may have these release-relevant tests carried out externally in accordance with Section 6 (3) ApBetrO. Preparation of pharmaceuticals to be kept in stock appears to be suitable and practicable for use in the diagnostic allergy work-up, whereas extemporaneous preparation is rarely practical due to the individual preparation for each patient and the associated complexities (see below) and costs. 

### Requirements of the Ph. Eur. for starting materials 

The monograph “Allergen products” (1063) [12] requires for source materials of all kinds that:

- they are obtained from qualified suppliers,
- they are defined, where possible, by their origin, nature, method of collection or production and pre-treatment,
- control methods and acceptance criteria relating to identity and purity are established,
- the source materials are stored under controlled conditions justified by stability data,
- when applicable, pesticides, heavy metals and residual solvents are limited (according to the principles defined in general chapters).

Microbial contamination of the native source material may be unavoidable and should be monitored. Foods must be of a quality suitable for human consumption. The origin of the foodstuff as well as its processing stage have to be stated. 

In addition to the general monograph “Allergen products” (1063) [12] source materials for skin test allergen production in pharmacies need to comply with the requirements of the appropriate individual monographs (where a relevant monograph exists), e.g., “Animal epithelia and outgrowths for allergen products” (2621) [14], “Mites for allergen products” (2625) [15] and “Moulds for allergen products” (2626) [16] (Table 1). 

There are three indicative scenarios for the production of skin prick test solutions in pharmacies:

- Scenario 1: A specific monograph is available in the pharmacopoeia, and the allergen starting material can be obtained commercially from certified manufacturers (example: storage mites).
- Scenario 2: A specific monograph is available in the pharmacopoeia, but the allergen starting material cannot be obtained commercially from a certified manufacturer (example: bovine hair).
- Scenario 3: A specific monograph is not available, and the allergen starting material cannot be obtained commercially from a certified manufacturer (example: wheat and rye flours).

As a matter of fact, each of the three scenarios has specific aspects for the production of the respective test allergens in the pharmacy. In order to examine the provided certification, starting material was obtained from the manufacturers Stallergenes Greer (Lenoir, NC, USA) and Allergon (Ängelholm, Sweden). 

For example 1 (scenario 1), the following applies: In addition to the identity testing of the starting material in accordance with ApBetrO, for storage mites as starting material the monograph “Mites for allergen products” (2625) [15] requires that foreign matter, defined as any particles that are not part of the mite culture, must be limited in the allergen source material. The water loss on drying (for dried material) and purity (when the source material is a purified fraction of the mite culture) have to be stated. In addition to the methods for identity testing in accordance with the monograph “Allergen products” [12], the monograph “Mites for allergen products” gives the possibility of using the macroscopic and microscopic morphological characteristics of the mites in comparison with a reference preparation to determine identity. Alternatively, the monograph states protein analysis (e.g., via the protein profile) or genetic identification [15]. 

Depending on availability, starting materials with certificates can currently be obtained, e.g., from Stallergenes Greer or Allergon. In the case of storage mites (Lepidoglyphus destructor, Tyrophagus putrescentiae, Acarus siro), the material can be obtained from both manufacturers (Table 2). Depending on the manufacturer, the information on the starting material in the certificate varies: 

In the available certificates for batches of starting material for storage mites (2021 – 2023), Stallergenes Greer provided general information on the product: allergen source including Latin name, item and lot number, collection date, condition, storage conditions, shelf life, processed following applicable current good manufacturing practice (cGMP) regulations as required under 21 CFR 680.1(b)^2^ [17]. 

^2^Applicable cGMP regulations as required under the Title 21 Code of Federal Regulations (CFR) 680.1(b) [17] provide additional standards on allergen products, e.g., criteria for source material, such as their identity and limit for detectable foreign materials. Detailed standards for allergen source materials from molds and animal epithelia are stated (Table 2). 

However, there was an information gap regarding internal tests carried out (reference is made to internal, non-monographed (non-compendial) methods, without indication of measured values or specified limits). This applies to the methods used to determine purity and identity as well as information on pesticides, heavy metals, solvent residues or microbial contamination. Information on water content or drying loss was also not stated in the certificate, nor was the cultivation method used (including medium and freedom from mold). If possible, these data should be requested from the manufacturer or, if not available there, obtained elsewhere in addition to the certificate. In order to limit starting material testing to confirmation of identity (according to Section 11 ApBetrO [10]), a certificate from a facility in accordance with Section 6 (3) ApBetrO is required. 

The certificates provided by Allergon for starting material batches of storage mites (obtained in 2020 and 2022) contained information on production and tests carried out, including target values, measured values, the test laboratory and methods, as well as images of the microscopic and gel electrophoretic analyses. Macroscopic and microscopic examinations were carried out on the mites, and the purity and absence of foreign mites or molds were determined. Furthermore, the average size of the mites, the water content and the total protein content were determined, and an SDS-PAGE was carried out. Information on the date of preparation and cultivation (e.g., temperature, humidity, culture vessel) was also included. The medium was treated with gamma radiation. Data on preparation (separation of mites, drying) and subsequent storage are also included. 

The certificate does not contain information on pesticides, heavy metals, solvent residues, microbial contamination, or the medium used, but the certificate does contain a final confirmation that the material complies with all requirements of the monograph “Mites for allergen products” (2625) [15]. Further information on the underlying quality assurance standards of the analytical laboratories is available on request. 

For example 2 (scenario 2), the following applies: In addition to the identity testing of the starting material in accordance with ApBetrO, for bovine hair as starting material the monograph “Animal epithelia and outgrowths for allergen products” (2621) [14] requires that foreign matter (here defined as mites, fleas, dirt and foreign epithelia) must be limited and the water content measured (if the product has been dried). In addition to the methods of identity testing stated in the monograph “Allergen products” [12], the monograph “Animal epithelia and outgrowths for allergen products” gives the possibility of identification via macroscopic and microscopic characteristics in comparison to a reference or via other methods, such as enzyme-linked immunosorbent assay (ELISA) or genetic identification. 

Bovine hair could not be obtained commercially as certified allergen starting material, but bovine epithelia could be purchased instead as certified starting material from the manufacturer Stallergenes Greer. In addition to the general information (allergen source including Latin name, article and batch number, collection date and method, storage conditions, shelf life) on the product processed following applicable cGMP regulations as required under 21 CFR 680.1(b) [17], a certificate on animal health, information on the collection and treatment (degreasing with acetone, drying) and a description of the macroscopic properties was provided. 

The certificate does not include precise information on the internal tests carried out (e.g., to determine purity and identity, as well as information on pesticides, heavy metals, solvent residues, or microbial contamination). In addition, the cGMP regulations required under 21 CFR 680.1(b) [17] state that the 1.0% limit for detectable foreign materials shall not apply for animal starting material. However, pelts, feathers, hairs and danders shall be collected in a manner that will minimize contamination of the source material. 

The certificate does not contain any information on the proportion of foreign matter, water content, or water loss on drying. If possible, these data should be requested from the manufacturer or, if not available, obtained additionally to the certificate. In order to limit starting material testing to confirmation of identity (according to Section 11 ApBetrO [10]), a certificate from a facility in accordance with Section 6 (3) ApBetrO is required. 

or example 3 (scenario 3), the following applies: For the source material wheat and rye flour, in addition to the obligation to verify identity in accordance with ApBetrO [10] only the monograph “Allergen products” (1063) applies [12] with the identity tests defined therein. 

Flours are currently not commercially available as a certified starting material. Coarse, lyophilized grain can be used in pharmacies, which was commercially available from Stallergenes Greer during the research project period. 

The following general and specific information is provided in the certificates for starting material batches (obtained in 2021 – 2023) of lyophilized wheat and rye grain processed following applicable cGMP regulations as required under 21 CFR 680.1(b) [17]: allergen sources including Latin name, item and batch number, date of manufacture, solvent used, condition, storage conditions, loss on drying, and shelf life. 

The certificate does not contain any information on internal tests carried out (e.g., to determine purity and identity) or on pesticides, heavy metals, solvent residues or microbial contamination. As grain is a foodstuff, the origin, processing, and suitability for human consumption must also be specified. If possible, these data should be requested from the manufacturer or, if not available, obtained additionally to the certificate. In order to limit starting material testing to confirmation of identity (according to Section 11 ApBetrO [10]), a certificate from a facility in accordance with Section 6 (3) ApBetrO is required. 

### Procedure for uncertified starting material without the possibility of substitution 

If it is not possible to switch to a certified starting material in cases of scenarios 2 and 3, the material must be completely tested for its suitability as a starting material in accordance with the monograph “Allergen products” (1063) [12] and any specific monographs that may apply. In order to limit starting material testing to confirmation of identity (according to Section 11 ApBetrO [10]), a certificate from a facility in accordance with Section 6 (3) ApBetrO is required. 

### Requirements of the Ph. Eur. for the end product (test allergen solution) 

The finished test allergen solution for skin testing must fulfill at least the following requirements according to the general monograph “Allergen products” (1063) [12]:

- Sterility, testing is performed in accordance with the monograph “Sterility” (2.6.1.) [18]
- Protein content between 80 and 120% of a defined value to be specified
- Protein profile comparable to an in-house reference preparation or a representative test allergen reference batch

### Practical preparation of skin prick test solutions from allergen starting materials in the pharmacy 

**Figure 1.**
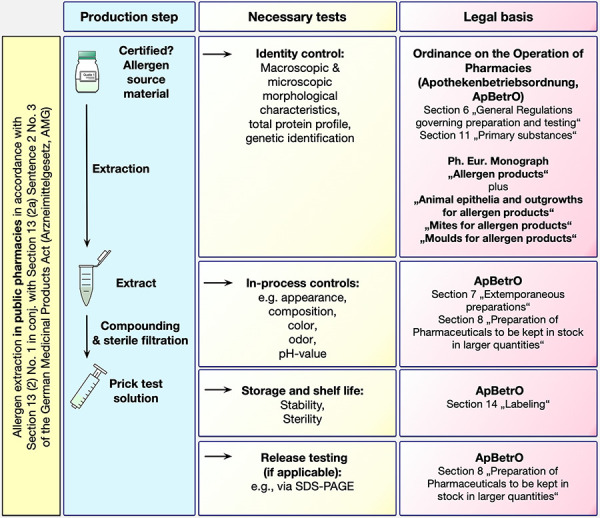
Legal basis for quality assurance in the preparation of skin prick test solutions in pharmacies, in accordance with AMG and ApBetrO. AMG = Medicinal Products Act; ApBetrO = Ordinance on the Operation of Pharmacies ; Ph. Eur = European Pharmacopoeia.

If allergen starting materials of sufficient quality are available, the preparation of skin prick test solutions can be carried out in the pharmacy in compliance with the above-mentioned legal provisions (Figure 1). According to Section 7 (1a) and Section 8 (1) ApBetrO [10] a written manufacturing instruction is initially drawn up for the respective process in the pharmacy; standardized and general third-party manufacturing instructions must be adapted to the respective pharmacy facility. 

The first step is to prepare an allergen extract from the allergen starting materials. To ensure the quality of the extracts, in-process controls in the pharmacy are crucial and necessary. These parameters include general organoleptics (appearance, texture, color, odor), temperature control, pH value, and checking the integrity of sterile filters used (according to Section 6 – Section 8 ApBetrO). 

Identity testing procedures are carried out on the allergen starting material or allergen extract before it is formulated into the finished skin prick test solution. According to legal requirements, the following test methods are suitable for this purpose:

- Total protein profile in direct comparison to a reference preparation (“in house reference preparation” or a representative batch of allergen source material), using for example isoelectric focusing, SDS-PAGE, immunoelectrophoresis, immunoblotting, liquid chromatography, or mass spectrometry [12]
- Macroscopic and microscopic morphological characteristics [15, 16]
- ELISA to determine the allergen content [14, 15, 16]
- Genetic identification [14, 15, 16]

The characterized allergen extract is then mixed with glycerol to form the finished skin prick test solution. The skin prick test solution must then be filtered sterile. A suitable filter material (hydrophilic for aqueous samples, low protein binding (e.g., cellulose acetate (CA) or polyethersulfone (PES), possibly also regenerated cellulose (RC) or polyvinylidene fluoride (PVDF)), as well as a suitable in-process control to assess the integrity of the filter must be observed. One possibility for this is the bubble point test [19] in which air must be compressible to a predefined volume without passing through the filter. 

Methodologically, sterile filtration can be carried out in the same way as the already established preparation of eye drops in pharmacies. Here, the solution is filtered directly into sterilized containers inside autoclave bags, which are then sealed while still in the bag. An analogous procedure for the preparation of skin prick test solutions could be the direct filling of sterile 1-mL fine-dose syringes as primary packaging material. 

Aqueous solutions without added preservatives may be susceptible to microorganisms despite their glycerol content [20]. For the final pharmacy product, according to Section 14 (1) No. 7 ApBetrO [10] the shelf life after filling and after opening must therefore be indicated. For classic pharmacy formulations, the recommendations of the DAC/NRF [19] are used for this purpose. 

The physical and chemical stability of the extracts must be taken into account here. Experience shows stability for several years under suitable storage conditions (S. Kespohl, personal communication). The volume of the fillings may have to be adjusted in the pharmacy to the shelf life after opening, even down to individual doses. Alternatively, a storage concept can be established in the pharmacy with final (renewed) sterile filtration shortly before dispensing. 

### Product testing of skin prick test solutions prepared in the pharmacy as preparations of pharmaceuticals to be kept in stock in larger quantities (“Defektur”) 

Finally, the end product of preparations of pharmaceuticals to be kept in stock in larger quantities (“Defektur”) must be subjected to an analytical test, which is suitable for identifying product-specific pharmaceutical risks (Section 8 ApBetrO) [10]. Taking into account the conditions in the pharmacy, an examination of the total protein profile in comparison to a defined reference batch using suitable methods appears necessary for test allergens. This is comparable to commercial test allergen production with comparison to a reference preparation (“in-house reference preparation”). 

A total protein content determination can be useful under certain circumstances as a test on the end product. If no protein-containing excipients are used, this usually correlates with the allergen content [8]. This can be carried out by external certified laboratories that fulfill the conditions of Section 6 (3) ApBetrO. According to Ph. Eur., the total protein content must be in the range of 80 – 120% of the declared total protein content [12]. Determining the total protein content directly in the pharmacy does not appear to be practicable due to the equipment required. 

With regard to the general question of whether, in the context of practice supplies^3^ (“Sprechstundenbedarf”), preparations manufactured in the pharmacy without reference to the patient’s name may be used or whether a patient-specific prescription is necessary, a court case was pending in Schleswig-Holstein using the example of “Fluorescein-Na (Inj.) 10%” – 5-mL syringes and the bowel cleansing powders “Pico-Citro Bowel Cleansing” and “Citro-2 Liter Bowel Cleansing” [21, 22]: On August 10, 2023, the Higher Administrative Court for the State of Schleswig-Holstein (3^rd^ Senate) ruled positively in favor of the use of extemporaneous preparations (“Rezeptur”) and preparations of pharmaceuticals to be kept in stock in larger quantities (“Defektur”) for the provision as practice supplies. The appeal has been allowed and the authority has filed an appeal with the Federal Administrative Court. The ruling could also have implications for the production of test allergens in pharmacies as practice supplies. 

^3^The term practice supplies denotes products such as pharmaceuticals, medical dressings, and similar materials that are regularly used on more than one patient with only a small portion of a single package in practice rooms or during home visits and/or must be available in emergencies. 

If an epidemiological study with new allergens to investigate the prevalence of sensitization (no clinical trial according to AMG) is intended, the preparation by a pharmacy is also conceivable, provided that the preparation for an epidemiological study is covered by the term “normal operation of a pharmacy” according to Section 13 (2) No. 1 AMG [1]. Otherwise, a manufacturing authorization according to Section 13 AMG would be necessary. The decision on the manufacturing authorization requirement is in the responsibility of the state authorities (“Länderbehörden”). 

## Discussion 

The existing legal requirements in Germany do allow the preparation of skin prick test solutions in pharmacies. For the majority of the 20 occupational allergen sources examined as examples (see article by Raulf & Kespohl in part 1 of the special issue [9]), potentially suitable allergen starting materials are commercially available and quality requirements are defined in the Ph. Eur. So far, however, no corresponding manufacture has taken place in pharmacies. This may be due to a lack of work instructions or standard operating procedures (SOPs), a lack of experience, a lack of demand, a remaining need for legal clarification, and/or unclear billing modalities. 

Various methods are used for the general analysis of formulation starting materials:

- By standard, the identity check can be carried out using the wet chemical method described in the respective monograph.
- Depending on the starting material, other physical processes are used, such as melting point determination, refractometry, or microscopy.
- The use of technical methods such as near-infrared spectroscopy is also permitted, which also allow a clear determination of identity.

With regard to the necessary identity testing of the allergen starting materials and the raw allergen extracts produced from them, however, no established routine method is currently available in a pharmacy. Near-infrared spectroscopy proved to be unsuitable in our investigations. In addition to the complex handling of the allergen starting material, a large number of reference spectra of different starting material batches would be necessary for the valid establishment of a reliable identification. The number of batches of allergen starting material required according to the device manufacturer exceeds the quantity currently available on the market by a factor of 5 – 10. According to our own investigations, testing the total protein profile by means of SDS-PAGE is instead expedient both in the identification of the starting material and in the final product testing (according to Section 8 (3) and (4) ApBetrO) in the pharmacy. However, the method is not a standard procedure in pharmacies and must therefore first be established before test allergen production. 

It remains to be legally clarified whether the use of certified animal and microbial allergen source materials requires a manufacturing authorization in accordance with Section 13 (1) No. 3 AMG [1] or whether the exemption permit for pharmacies (Section 13 (2) No. 1 in conjunction with Section 13 (2a) sentence 2 No. 3 AMG) also covers this step (extraction of the certified allergen source material in the pharmacy). This question arises from a ruling by the Federal Administrative Court (Bundesverwaltungsgericht = BVerwG) from September 3, 2020 (BVerwG 3 C 10.18) [23] on the production of “fresh cells” from animal organs for later injection into humans (production by a physician without a manufacturing authorization, due to the Physicians’ privilege according to Section 13 (2b) sentence 1 AMG), from which implications for the production of test allergens in pharmacies (based on Section 13 (2) No. 1 in conjunction with Section 13 (2a) sentence 2 No. 3 AMG) from animal or microbial starting materials could be derived. In the ruling there, a distinction was made between the manufacture of a medicinal product and the manufacture of an active ingredient, and the above-mentioned physicians’ privilege was only applied to the manufacture of a medicinal product. In the production of “fresh cells” from animal organs, the production from the starting material through the active ingredient stage to the finished medicinal product takes place in one go, similar to the production of test allergens, which results in the analogy to be clarified legally. Fundamentally, however, the manufacturing competencies transferred to pharmacies under Section 13 (2) No. 1 AMG are more extensive than those conferred on physicians (e.g., manufacturing for more than one patient is permitted); and the authorization to manufacture test allergens is explicitly mentioned in Section 13 (2a) sentence 2 No. 3 AMG. 

The general need for a certificate in accordance with Section 6 (3) ApBetrO must also be clarified [10] for native allergen starting material prior to further processing in the pharmacy. In the opinion of the authors, allergen starting material is material prior to the active ingredient stage [11], which is why no general requirement for certification in accordance with Section 6 (3) ApBetrO can be derived from the ApBetrO. 

In addition, the current prohibition of brokering transactions (“Makelverbot”) according to Section 11 (1) ApoG [13] must be observed: permit holders and pharmacy staff may not execute legal transactions or enter into agreements with physicians or other individuals concerned with the treatment of health disorders involving the preferential supply of certain pharmaceuticals, the referral of patients, or the assignment of prescriptions. Exceptions to this currently only exist for cytostatic preparations (Section 11 (2) ApoG) [13]. Direct allocation of prescriptions to selected pharmacies should therefore be avoided. In principle, the preparation of skin prick test solutions should be possible in any interested pharmacy. 

Another point to be clarified is the pricing of the skin prick test solutions produced, as the normal pharmacy billing rates do not cover the manufacturing costs. 

## Conclusion 

The preparation of skin prick test solutions by public pharmacies is a legally permissible alternative to ensure supply in the event of a continuing shortage of authorized commercial skin test allergens for occupational allergies. Suitable certified allergen starting materials are commercially available for numerous occupational allergen sources, and their quality requirements are defined in the Ph. Eur. Once the remaining legal issues have been clarified, the pharmacy manufacture of skin prick test solutions for rare allergen sources could make an important contribution to closing the existing diagnostic gap. 

## Disclaimer 

The views expressed in this review are the personal views of the authors and should not be understood or quoted as being made on behalf of, or reflecting the position of, the relevant national competent authorities, the European Medicines Agency or any of its committees or working parties. 

## Acknowledgments 

The authors would like to thank Ms. Marion Frech (PEI, Section SBD 4 Legal Affairs) and Dr. Frank Führer (PEI, Section ALG 3 Allergens Product Testing) for reviewing sections of the manuscript. 

## Authors’ contributions 

VM and MR conceptualized the article, JR, SSchü and VM wrote and revised the manuscript, all authors discussed the content, commented the manuscript and approved its final version. 

## Funding 

The work was supported in part by DGUV research funding (research project FB 0317a – Quality control of the diagnostics for occupational type I allergies). 

## Conflict of interest 

R. Jost: The present work was carried out in (partial) fulfillment of the requirements for the degree “Dr. rer. biol. hum.” at the Friedrich-Alexander-University Erlangen-Nuremberg (FAU). 

All other authors state that there is no conflict of interest. 

**Table 1.** Requirements for allergen products / applicable tests according to the monographs of the European Pharmacopoeia, depending on the allergen source (selection according to a needs survey carried out amongst statutory accident insurance institutions, see article by Raulf & Kespohl in part 1 of the special issue [9]).

**Allergen source**	**Requirements for the source material according to the monograph (Ph. Eur.)**	**Requirements for the final product according to the monograph (Ph. Eur.)**
Wheat (flour, grain)	“Allergen products (01/2022:1063)” – Obtained from qualified suppliers – Defined, where possible, by their origin, nature, method of collection or production, and pretreatment – Establishment of control methods and acceptance criteria relating to identity and purity – Storage under controlled conditions, justified by stability data; ensuring a consistent composition from batch to batch – When applicable, pesticides, heavy metals, and residual solvents are limited – Microbial contamination should be monitored – For foods: suitable quality for human consumption, the origin, and the processing stage is stated	“Allergen products (01/2022:1063)” Protein content (80 – 120% of the stated content) – Protein profile (corresponds to that of the in-house reference preparation) – Water/loss on drying (for freeze-dried products) – Sterility (parenteral preparations, eye preparations, preparations for inhalation or preparations for skin testing) – Microbial contamination (for non-sterile allergen products)
Rye (flour, grain)		
α-amylase		
Glucoamylase		
Spruce wood		
Beech wood		
Latex		
Phytase		
Fish (cod/salmon) and a freshwater fish		
Shellfish (shrimp)		
*Lepidoglyphus destructor*	“Mites for allergen products (01/2017:2625)” In addition to the monograph “Allergen products” – Foreign matter, defined as any particles that are not part of the mite culture; detected using a suitable microscopic method, with a limit based on historical data – Water / loss on drying (for dried material) – Purity (when the source material is a purified fraction of the mite culture)	
*Tyrophagus putrescentiae*		
*Acarus siro*		
Cattle hair/epithelia	“Animal epithelia and outgrowths for allergen products (01/2017:2621)” In addition to the monograph “Allergen products” – Foreign matter, defined as vermin (e.g., mites and fleas), dirt, and foreign animal epithelia and outgrowths; determination by microscopic examination, ELISA, visual inspection and/or tactile inspection, must be below a predefined and justified limit – Water/loss on drying (for dried material)	
Mouse hair/urine		
Rat hair/urine		
*Aspergillus fumigatus*	“Moulds for allergen products (01/2017:2626)” In addition to the monograph “Allergen products” – Foreign species, their absence is determined by macroscopic and microscopic examination – Water/loss on drying (for dried material) – Mycotoxins, their content is determined	
*Penicillium chrysogenum*		
Mold (as a moisture indicator, e.g., *Aspergillus versicolor*)		
*Stachybotris chartarum*		

**Table 2.** Requirements of the European Pharmacopoeia (monographs “Allergen products” (1063), “Animal epithelia and outgrowths for allergen products” (2621) and “Mites for allergen products” (2625)) compared to requirements of current good manufacturing practice regulations according to 21 CFR 680.1(b); information in these certificates on starting material batches (from Stallergenes Greer 2021 – 2024 with reference to 21 CFR 680.1(b)) and Allergon 2020 – 2022 with reference to European Pharmacopoeia).

**Ph. Eur. requirements **	**21 CFR 680.1(b)**	**Allergon – storage mites**	**Stallergenes Greer – storage mites**	**Stallergenes Greer – animal epithelia**	**Stallergenes Greer – cereal grain**
Obtained from qualified suppliers	Listing of source materials and suppliers: Each licensed manufacturer shall initially list with the Director, Center for Biologics Evaluation and Research, the name and address of each of the manufacturer’s source material suppliers. The listing shall identify each source material obtained from each source material supplier. The licensed manufacturers shall update the listing annually to include new source material suppliers or to delete those no longer supplying source Materials. Nonlicensed source material suppliers are exempt from drug registration.	√	√	√	√
Defined, where possible, by their origin, nature, method of collection, or production and pretreatment	Only specifically identified allergenic source materials that contain no more than a total of 1.0 percent of detectable foreign materials shall be used in the manufacture of Allergenic Products.	√	√	√	√
Establishment of control methods and acceptance criteria relating to identity and purity	n.s.	√	n.s.	n.s.	n.s.
Storage under controlled conditions, justified by stability data; ensuring a consistent composition from batch to batch	n.s.	√	√	√	√
When applicable, pesticides, heavy metals, and residual solvents are limited	n.s.	n.s.	n.s.	n.s.	n.s.
Microbial contamination should be monitored	n.s.	n.s.	n.s.	n.s.	n.s.
**For foods: ** Suitable quality for human consumption, origin, and the processing stage is stated	Mammals and birds, subject to inspection by the U.S. Department of Agriculture at the time of slaughter and found suitable as food, may be used as a source material, and the requirements of paragraph (b)(3) (i) through (iv) of this section do not apply in such a case. Reporting obligations and obligations to store dead animals remain unchanged.	n.a.	n.a.	n.a.	n.s.
**For mites/epithelia: ** Limit on foreign matter	No more than a total of 1.0 percent of detectable foreign materials **Not applicable for molds and animals described**	√	(√) Not specified, but follows from 21 CFR 680.1(b)	n.s.	n.a.
F**or mites/epithelia:** Water/loss on drying (for dried material)	n.s.	√	n.s.	n.s.	n.a.
**For mites: ** Purity (when the source material is a purified fraction of the mite culture)	n.s.	√	n.s.	n.a.	n.a.
**For mites** Method of mite cultivation is described, critical parameters are controlled and monitored	n.s.	√	n.s.	n.a.	n.a.
For mites: Unless otherwise justified, the culture medium is selected to avoid the presence of materials with potential allergenicity	n.s.	(√) Free of mold No indication of the medium	n.s.	n.a.	n.a.
**For mites: ** Inactivation by methods, which are qualified capable to maintain allergenic properties	n.s.	√ Gamma rays	√ Freezing	n.a.	n.a.
**For epithelia:** Healthy animals, confirmed by a responsible veterinarian or another competent person, skin visibly clean and intact, and the animal have not been recently treated with preparations for cutaneous application	Maintenance by competent personnel in facilities or designated areas that will ensure adequate care, only animals in good health and free from detectable skin diseases shall be used as a source material for Allergenic Products, determination of good health shall be made by a licensed veterinarian or a competent individual under the supervision and instruction of a licensed veterinarian	n.a.	n.a.	√	n.a.
For epithelia: Exact species and/or variety is stated	Stated above: Only specifically identified allergenic source materials shall be used in the manufacture of Allergenic Products	n.a.	n.a.	√	n.a.
For epithelia: Collection without injuring the skin, prevention of cross-contamination, source material collection is specified	Pelts, feathers, hairs and danders shall be collected in a manner that will minimize contamination of the source material	n.a.	n.a.	√	n.a.
n.s.	**For mammals/birds:** Immunization against tetanus	n.a.	n.a.	(√) Not specified, but implicit in stated compliance to 21 CFR 680.1(b)	n.a.
n.s.	**For mammals/birds:** Reporting of cases of actual or suspected infection with foot and mouth disease, glanders, tetanus, anthrax, gas gangrene, equine infectious anemia, equine encephalomyelitis, or any of the pock diseases	n.a.	n.a.	(√) Not specified, but implicit in stated compliance to 21 CFR 680.1(b)	n.a.
n.s.	**For mammals/birds:** Dead animals may be used as source material in the manufacture of Allergenic Products, provided that the carcasses shall be frozen or kept cold until the allergen can be collected, or shall be stored under other acceptable conditions so that the postmortal decomposition processes do not adversely affect the allergen; when alive, the animal met the applicable requirements, previously mentioned	n.a.	n.a.	(√) Not specified, but implicit in stated compliance to 21 CFR 680.1(b)	n.a.

n.s. = not specified; n.a. = not applicable; Ph. Eur = European Pharmacopoeia.
